# Supplementary material for: Estrogen and Progesterone Receptors Are Dysregulated at the BPH/5 Mouse Preeclamptic-Like Maternal–Fetal Interface
Source: Biology (Basel). 2024 Mar 16;13(3):192. doi: 10.3390/biology13030192 (PMC10967993; doi:10.3390/biology13030192)
Supplement: Supplementary file 1 [file biology-13-00192-s001.zip › biology-2868664-supplementary.pdf]

*Research Article*

# Estrogen and Progesterone Receptors are Dysregulated at the BPH/5 Mouse Preeclamptic-Like Maternal–Fetal Interface

**Viviane C. L. Gomes [1], Bryce M. Gilbert [1], Carolina Bernal [1], Kassandra R. Crissman [2], and Jenny L. Sones [3*]**

[1] Department of Small Animal Clinical Sciences, Michigan State University College of Veterinary Medicine, East Lansing, MI 48824, United States.
[2] Department of Veterinary Clinical Sciences, Louisiana State University School of Veterinary Medicine, Baton Rouge, LA 70803, United States.
[3] Colorado State University Equine Reproduction Laboratory, Fort Collins, CO 80521, United States.
* Correspondence: jenny.sones@colostate.edu

## Supplementary Material

**Table S1**. Forward and reverse Mus musculus-specific primer sequences used in qRT-PCR

| Gene | Primer Sequence |
|---|---|
| *18S* | F: GTAACCCGTTGAACCCCATT<br>R: CCATCCAATCGGTAGTAGCG |
| *Esr1* | F: AAT TCT GAC AAT CGA CGC CAG<br>R: GTG CTT CAA CAT TCT CCC TCC TC |
| *Esr2* | F: CGG TCT GTC TGA ATG TGG TCA CTG<br>R: TTG CCT TGG TCA TGG TAT CGC TTC |
| *Pr-A/B/C* | F: GAC ACT GGC TGT GGA ATT TCC<br>R: CCA GGA TCT TGG GCA ACT G |
| *Pr-A/B* | F: CTG GAG ACC GAG GGC TCT<br>R: CCA GTG CTC GAG GTT TGC TC |
| *Pr-B* | F: GTG GAG GGC GCT TTC TCT G<br>R: TCT GCC TCC CTC CCT ATG AGT |
